# Development and Validation of a Novel Risk Score for All-Cause Mortality Risk Stratification Prior to Permanent Pacemaker Implantation in Octogenarians or Older

**DOI:** 10.3390/medicina59081499

**Published:** 2023-08-21

**Authors:** Hsuan-Ching Lin, Ming-Jui Hung, Chao-Hung Wang, Tien-Hsing Chen, Wei-Siang Chen, Chi-Wen Cheng

**Affiliations:** 1 Division of Cardiology, Department of Internal Medicine, Chang Gung Memorial Hospital, 222 Mai Chin Road, Keelung 204201, Taiwan; 2 Chang Gung University College of Medicine, No. 259, Wenhua 1st Rd., Guishan Dist., Taoyuan City 333323, Taiwan

**Keywords:** frail elderly, octogenarian, pacemaker, survival

## Abstract

*Background and Objectives*: The demand for permanent pacemaker (PPM) implantation for extremely old patients is increasing. Prior to implanting PPMs, life expectancy evaluation is essential but difficult. We aimed to develop and validate a scoring system for all-cause mortality risk stratification prior to PPM implantation in patients aged ≥80. *Materials and Methods*: A total of 210 patients aged ≥80 who received PPM implantation were included. Multivariable analysis was performed to assess the effects of different variables on all-cause mortality in a derivation cohort (*n* = 100). We developed the MELODY score for stratifying all-cause mortality prior to PPM implantation and tested the scoring system in a validation cohort (*n* = 102). *Results*: After 4.0 ± 2.7 years of follow-up, 54 patients (54%) had died. The 0.5-, 1- and 2-year all-cause mortality rates were 7%, 10% and 24%, respectively. The MELODY score based on body mass index <21 kg/m^2^ (HR: 2.21, 95% CI: 1.06–4.61), estimated glomerular filtration rate <30 mL/min/1.73 m^2^ (3.35, 1.77–6.35), length of hospitalization before PPM implantation >7 days (1.87, 1.02–3.43) and dyspnea as the major presenting symptom (1.90, 1.03–3.50) successfully distinguished patients at high risk of mortality. Patients with MELODY scores ≥3 had a higher risk of mortality compared to those with MELODY scores <3 (8.49, 4.24–17.00). The areas under the receiver operating characteristic curves in predicting 0.5, 1 and 2 years mortality rates were 0.86, 0.81 and 0.74, respectively. The predictive value of the model was confirmed in a validation cohort. *Conclusions*: The novel scoring system is a simple and effective tool for all-cause mortality risk stratification prior to PPM implantation in patients aged ≥80.

## 1. Introduction

Worldwide, populations are aging rapidly. The number of people above age 80 is the fastest-growing group in all populations. Globally, in 1990, there were only 54 million people aged 80 and older. That number nearly tripled to 143 million by 2019. In the future, the number of people aged 80 or over is projected to nearly triple again to 426 million by 2050 [1]. As bradycardia and conduction disorders are commonly associated with degenerative fibrosis [2,3], the need for permanent pacemaker (PPM) implantation for extremely old patients with multiple comorbidities and relatively short life expectancies has increased [4]. In fact, roughly one-third of all patients receiving PPM implantation over the past 20 years were aged 80 and older, and that percentage has been increasing since 2002 [5,6,7].

Although PPM implantations are commonly performed heart procedures and not associated with high procedural risk in most patients [2], studies have shown that more than 10% of patients aged 80 and older who received PPM implantation died of non-PPM related causes within a year after the procedure [6,8,9,10]. Despite the fact that the decision to place a PPM is most likely going to be taken based on the evidence of conduction abnormalities, current practice guidelines recommend that patients and clinicians share the decision to implant PPM based not only on clinical indications but also on patients’ life span and overall prognosis [2,3]. Life expectancy evaluation prior to implanting PPM in extremely old patients is therefore necessary.

Few studies to date have focused on pre-PPM implantation evaluation of life expectancy for patients aged 80 and older, and there has been no general agreement on this issue. Recently, Balla et al. found that comorbidities were associated with worse survival after PPM implantation in patients aged 80 and older after a median follow-up of two years. They developed a scoring tool to identify patients with high three-month mortality risk after PPM implantation [9]. In a previous study, in addition to comorbidities, we included and analyzed other clinical characteristics of patients aged 80 or over who received PPM implantation. In that study, predictors of long-term survival included longer length of hospital stay before PPM implantation (LOS-B), estimated glomerular filtration rate (eGFR) <30 mL/min/1.73 m^2^, body mass index (BMI) <21 kg/m^2^ and dyspnea as the major presenting symptom [10]. The aim of this study was to develop a scoring system based on these clinical variables. Further, we validated our scoring system in another cohort. Effective risk stratification of all-cause mortality prior to PPM implantation for extremely old patients is informative, not only for the clinical decision-making process or for identifying patients in need of more intensive monitoring and therapy but also for future studies tackling this issue.

## 2. Methods

### 2.1. Study Population and Definition

**Figure 1:**
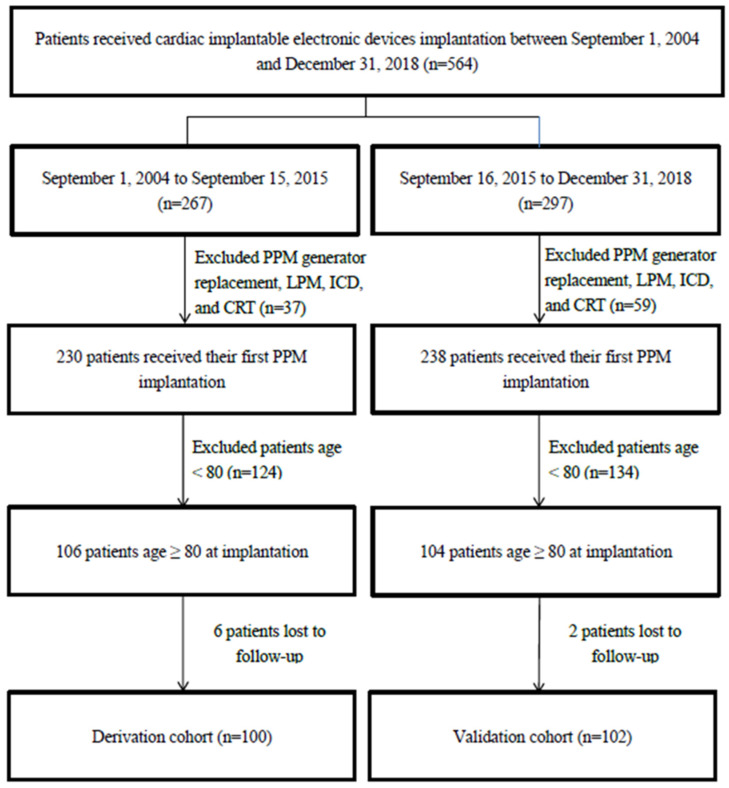
Flow chart for patient inclusion. CRT, cardiac resynchronization therapy; ICD, implantable cardioverter defibrillator; LPM, leadless pacemaker; PPM, permanent pacemaker.

We retrospectively included consecutive patients aged 80 and older who underwent pacemaker implantation according to international guidelines [2,3] in Keelung Chang Gung Memorial Hospital between 1 September 2004 and 31 December 2018 for analysis. Exclusion criteria were: first, patients receiving a pacemaker generator replacement (not their first PPM); second, patients who received leadless pacemakers, implantable cardioverter defibrillators, or cardiac resynchronization therapy; third, patients less than 80 years of age. Patients who received PPM implantation between 1 September 2004 and 15 September 2015 were included in the derivation cohort with complete follow-up by 15 December 2017. The validation cohort consisted of patients who received PPM implantation between 16 September 2015 and 31 December 2018, and their follow-up was complete by 8 October 2021 (Figure 1). Dyspnea was defined as the main symptom at presentation when the patient’s chief complaint was breathing discomfort rated as grade 2 or higher on the Modified Medical Research Council scale, a common tool for assessing dyspnea severity [11,12]. The details of the derivation cohort were described in our prior study [10].

### 2.2. Patient Follow-Up and Main Outcome of Interest

We obtained the survival status and causes of death from chart review. If patients were lost to follow-up from the pacemaker outpatient department for more than three months, we contacted them by telephone to find out their vital status and current condition. The main outcome of interest was all-cause mortality. This study was designed and conducted in accordance with the principles of the Declaration of Helsinki and was approved and monitored by the Institutional Review Board of Chang Gung Medical Foundation.

### 2.3. Statistical Analysis

For normally distributed continuous variables, we presented the data as mean ± standard deviation, as medians with first and third quartiles for variables with skewed distribution, and as numbers with percentages for categorical variables. Data were compared by independent sample t and Chi-square tests when appropriate. To assess the effects of different variables on survival, we performed Cox regressions with backward selection analysis. Variables with a *p* value of <0.05 in univariate analysis were included in the multivariable analysis. We also calculated hazard ratios (HRs) and 95% confidence intervals (CIs). The variables that remained statistically significant in the multivariable analysis were used as elements of the risk-scoring system. For purposes of clinical utility, the beta coefficients for these elements were converted to integer values in the risk-scoring system. Patients’ survival following PPM implantation was plotted as Kaplan–Meier curves, and we used the log-rank test to determine statistical significance. Receiver operating characteristic (ROC) curve analysis and Youden’s index were used to identify cutoff values. A *p* value of less than 0.05 was considered statistically significant. Statistical analyses were performed using SPSS Statistics 17.0 (SPSS Inc., Chicago, IL, USA, Released 2008. SPSS Statistics for Windows, Version 17.0. Chicago: SPSS Inc.).

## 3. Results

### 3.1. Study Patients

In total, 564 patients received cardiac implantable electronic devices implantation between 1 September 2004 and 31 December 2018. After excluding patients who received pacemaker generator replacement (not their first PPM), leadless pacemaker, implantable cardioverter defibrillators and cardiac resynchronization therapy, the number of patients receiving their first PPM implantation between 1 September 2004 and 15 September 2015 and between 16 September 2015 and 31 December 2018 were 230 and 238, respectively. The first group was the derivation cohort, and the second was used for validation (Figure 1). Of the 230 patients receiving PPM implantation in the first group, 106 (46.1%) were aged 80 and older. Of those 106 patients, 100 (94.3%) were followed up completely and included in the derivation cohort. Of the 238 patients receiving PPM implantation between 16 September 2015 and 31 December 2018, 104 (43.7%) were aged 80 and older. Of these 104 patients, 102 (98.1%) were followed completely and included in the validation cohort for later analysis.

### 3.2. Patient Characteristics

The patients’ baseline characteristics are shown in Table 1. The median age at implantation was 84.5 and 85.0 for the derivation and validation cohorts, respectively. The percentage of male patients was higher in the derivation than in the validation cohort (52.0 versus 37.3%, *p =* 0.04). In the derivation cohort, one-half of the patients presented with dyspnea, and others presented with dizziness (26%) and syncope or near syncope (24%). In the validation cohort, the majority presented with dizziness (51%), followed by dyspnea (32.4%) and syncope or near syncope (16.7%).

The patients had multiple comorbidities, including hypertension, diabetes mellitus, coronary artery disease, valvular heart disease, cerebral vascular accident, chronic obstructive pulmonary disease and atrial arrhythmia. Compared with the validation cohort, the derivation cohort had more hypertensive (88.0 versus 75.5%, *p =* 0.02) and fewer atrial arrhythmia patients (39.0 versus 52.9%, *p =* 0.04). The eGFR and BMI values did not differ statistically between the two cohorts, but the percentage of patients with BMI < 21 kg/m^2^ was lower in the derivation than in the validation cohort (17.0 versus 30.4%, *p* = 0.03).

Compared with the validation cohort, a higher percentage of patients in the derivation cohort were receiving PPM implantation due to atrioventricular conduction dysfunction (AVCD) (70.0 versus 42.2%, *p* < 0.001), and a lower percentage received dual chamber PPM implantation (80.0 versus 91.2%, *p =* 0.02). All of the patients who received single chamber PPM implantation received VVI or VVIR mode pacing, regardless of the cohort.

The length of hospital stay before permanent pacemaker implantation (LOS-B) was longer in the derivation than in the validation cohort (6 (3; 11) versus 3 (1; 7) days, *p* = 0.001). Prolonged LOS-B in the derivation cohort was due to systemic infection with 35 patients (35%) in need of parenteral antibiotic treatment, 21 (21%) with acute kidney injury resulting in electrolyte imbalance and fluid overload, 12 patients (12%) needing cardiac catheterization to estimate coronary artery disease, 9 (9%) in respiratory failure with mechanical ventilator support, 7 (7%) with gastrointestinal tract bleeding, and significant coronary artery disease needing intervention in 5 patients (5%).

### 3.3. Major PPM Implantation-Related Complications

Altogether, after PPM implantation, two patients (1.0%) (one from the derivation cohort and the other from the validation cohort) developed pneumothorax, and two patients (1.0%) developed pericardial effusion (one from each cohort). Both of the patients who developed pneumothorax were managed successfully with pigtail catheter placement. For the two patients who developed pericardial effusions, one from the derivation cohort recovered spontaneously without intervention, while the other patient from the validation cohort received pericardiocentesis and ventricular lead revision. No other significant complications, such as pacing system infection or procedure-related mortality, occurred in either cohort.

### 3.4. Causes of Death

**Figure 2:**
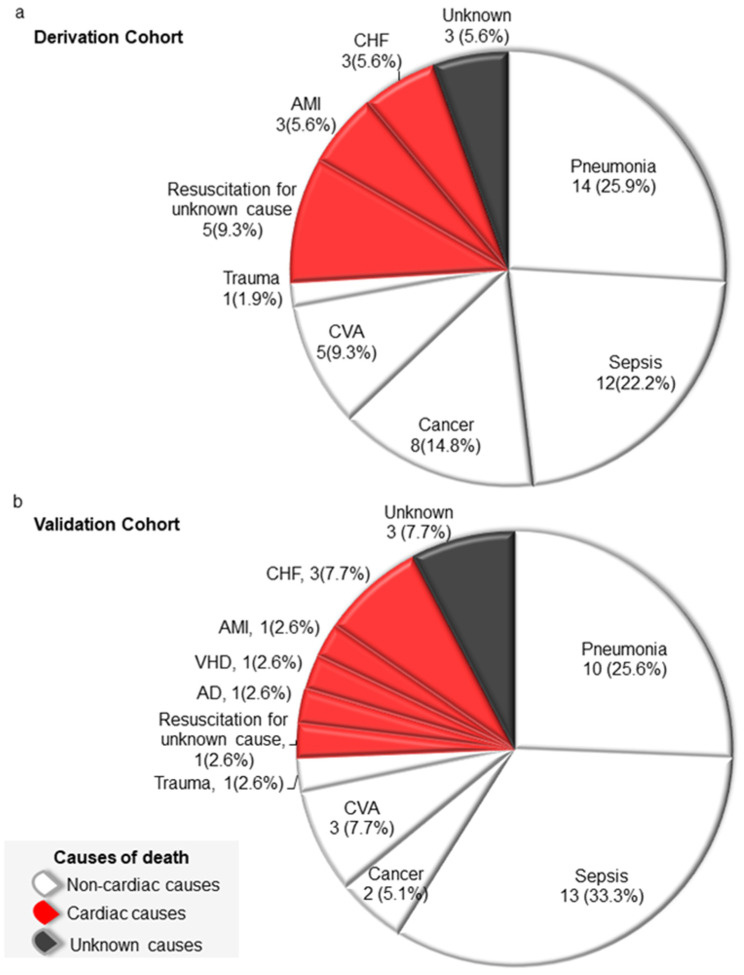
Causes of death and mortality rates in patients receiving permanent pacemaker implantation aged 80 and older after follow-up of 4.0 ± 2.7 and 2.8 ± 1.3 years in the derivation (**a**) and validation (**b**) cohorts, respectively. No deaths were related to permanent pacemaker implantation. AMI, acute myocardial infarction; AD, aortic dissection; CVA, cerebral vascular accident; CHF, congestive heart failure; VHD, valvular heart disease.

The follow-up duration was 4.0 ± 2.7 years for the derivation and 2.8 ± 1.3 years for the validation cohort. By the end of the study, 54 patients (54.0%) in the derivation cohort and 39 patients (38.2%) in the validation cohort had died. Of the patients who died in both cohorts, 74% of them died of non-cardiac causes, around one-fifth of them died of cardiac causes, and others died of unknown causes (Figure 2). Among patients who died of non-cardiac causes, the most common cause was pneumonia (35%) for the derivation cohort and sepsis (44.8%) for the validation cohort. No deaths were related to PPM implantation.

### 3.5. Predictors of All-Cause Mortality Prior to PPM Implantation

In Cox univariate analysis, variables associated with significantly lower cumulative survival rate included: dyspnea as the major presenting symptom, eGFR < 30 mL/min/1.73 m^2^, BMI < 21 kg/m^2^, atrioventricular conduction dysfunction as the indication for PPM implantation and longer LOS-B (Table 2). The cutoff value of LOS-B associated with all-cause mortality was set at seven days, based on the ROC curve analysis and Youden’s index. Cox multivariable analysis showed that patients with the following characteristics were associated with worse long-term survival: dyspnea as the major presenting symptom (HR = 1.90, 95% CI = 1.03–3.50, *p* = 0.03), eGFR < 30 mL/min/1.73 m^2^ (HR = 3.35, 95% CI = 1.77–6.35, *p* < 0.001), BMI < 21 kg/m^2^ (HR = 2.21, 95% CI = 1.06–4.61, *p* = 0.03) and LOS-B > 7 days (HR = 1.87, 95% CI = 1.02–3.43, *p* = 0.04).

### 3.6. Development of a Scoring System for All-Cause Mortality Risk Stratification Prior to PPM Implantation

We developed a scoring system based on the beta coefficients of significant variables identified in the multivariable analysis. To simplify the scoring system for clinical use and because the beta coefficient for eGFR (<30 mL/min/1.73 m^2^) was near twice that of BMI (<21 kg/m^2^), LOS-B (>7 days) and dyspnea as the major presenting symptom (Table 2), we assigned two points to the item eGFR (<30 mL/min/1.73 m^2^) and one point for the other items (Table 3). The sum of all points for these four items constituted the MELODY score, which ranged between 0 and 5 depending on the number of presenting characteristics and points for each patient.

### 3.7. Long-Term Mortality Risk Stratified by the MELODY Score

**Figure 3:**
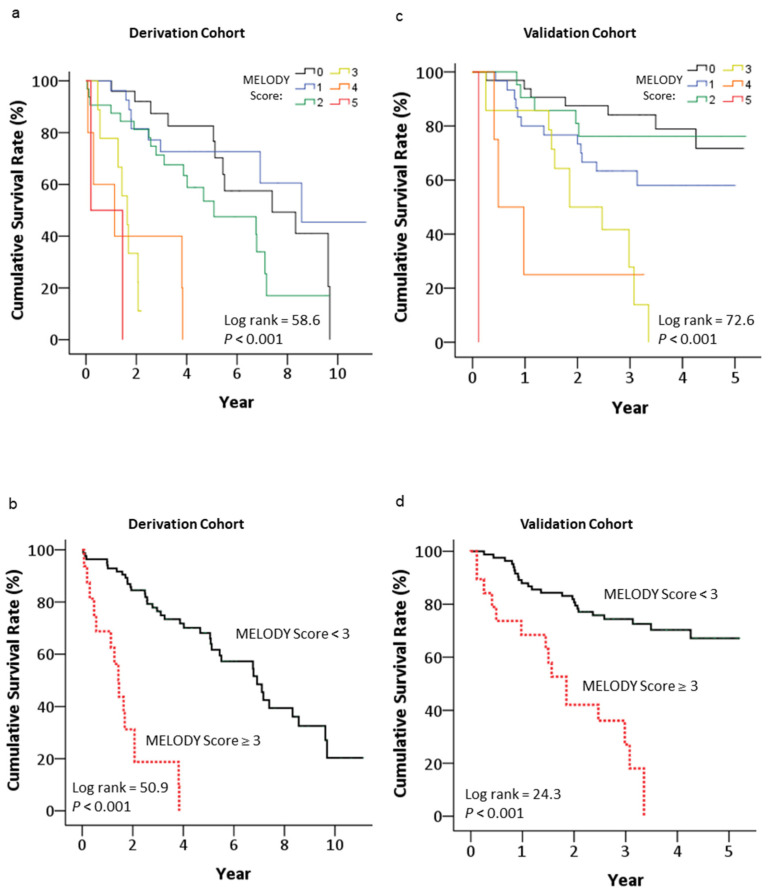
Kaplan–Meier survival curves for all-cause mortality after permanent pacemaker implantation in patients aged 80 and older categorized by MELODY scores of 0, 1, 2, 3, 4 and 5 in the derivation (**a**) and validation (**c**) cohorts and categorized by MELODY scores <3 and ≥3 in the derivation (**b**) and validation (**d**) cohort.

For patients with MELODY scores of 0, 1, 2, 3, 4 and 5 in the derivation cohort, the median survival time after PPM implantation was 7.4, 8.6, 5.1, 1.6, 1.1 and 0.2 years, respectively. Survival time decreased progressively as the scores increased (overall log rank = 58.6, *p* < 0.001) (Figure 3a). To predict mortality, the cutoff value for the score was set at three, based on Youden’s index. Patients with MELDOY scores ≥3 had a significantly higher risk of mortality compared to those with MELODY scores <3 (log rank = 50.9, *p* < 0.001, HR = 8.49, 95% CI = 4.24–17.00, *p* < 0.001) (Figure 3b). The median survival time after PPM implantation for patients with MELDOY scores ≥3 and <3 in the derivation cohort was 1.43 years (95% CI = 1.08–1.78) and 6.92 years (95% CI = 5.34–8.49), respectively.

### 3.8. The Power of MELODY Scores for Predicting All-Cause Mortality 0.5, 1 and 2 Years after PPM Implantation

**Figure 4:**
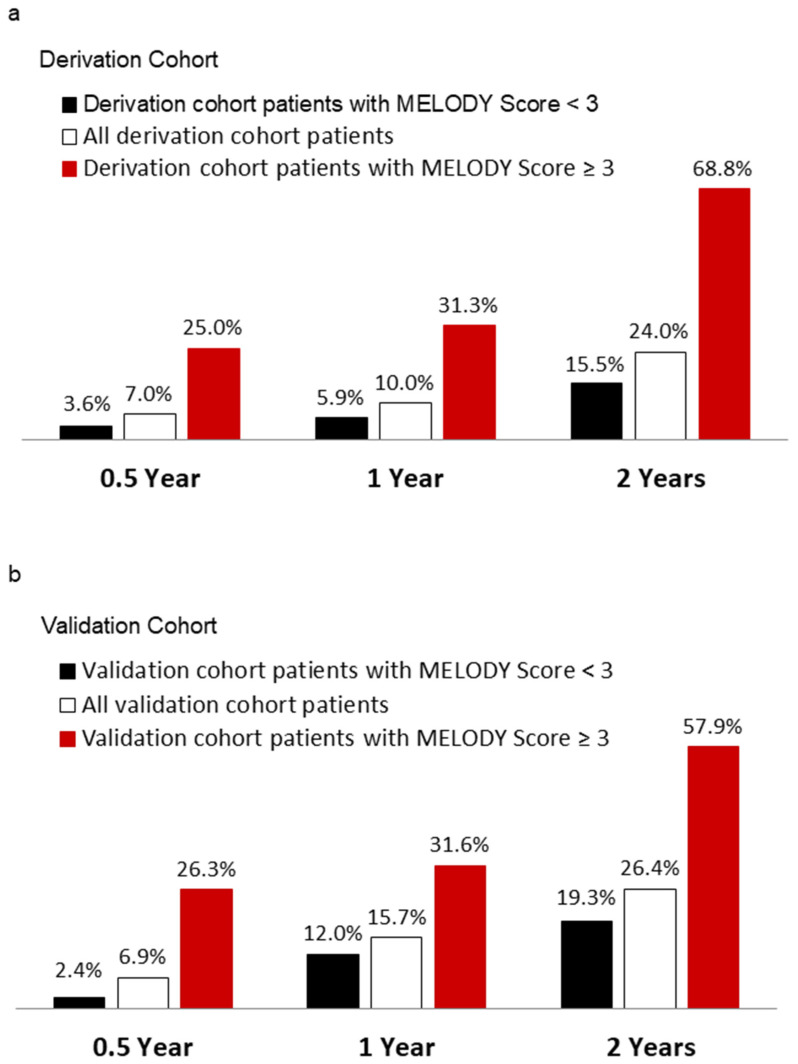
All-cause mortality rates at 0.5, 1 and 2 years after PPM implantation in patients aged 80 and older were categorized by MELODY scores <3 and ≥3 in the derivation (**a**) and validation (**b**) cohorts.

In order to test the power of MELODY scores for identifying patients with extremely short life expectancy after PPM implantation, we further analyzed how effectively MELODY scores predicted mortality rates 0.5, 1 and 2 years after PPM implantation. The mortality rates for all patients in the derivation cohort at 0.5, 1 and 2 years after PPM implantation were 7.0%, 10.0% and 24.0%, respectively (Figure 4a). For patients with MELODY scores <3 in the derivation cohort, mortality rates after PPM implantation were 3.6%, 5.9% and 15.5% at 0.5, 1 and 2 years, respectively. In contrast, for patients with scores ≥3 in the derivation cohort, mortality rates increased to 25.0%, 31.3% and 68.8% at 0.5, 1 and 2 years after PPM implantation, respectively. An ROC analysis of MELODY scores in predicting 0.5-, 1- and 2-year mortality rates after PPM implantation showed a significant predictive area under the curve values of 0.86, 0.81 and 0.74, respectively (Table 4).

### 3.9. Validation of the MELODY Score

The prognostic value of the MELODY score was further tested in the validation cohort. The survival time after PPM implantation in the validation cohort decreased progressively as scores increased (overall log rank = 72.6, *p* < 0.001) (Figure 3c). Patients with MELODY scores ≥3 had a significantly higher risk of mortality compared to those with scores <3 (log rank = 24.3, *p* < 0.001, HR = 4.61, 95% CI = 2.37–8.98, *p* < 0.001). The median survival time for patients with MELODY scores ≥3 was 1.85 years (95% CI = 1.36–2.34), in contrast to the median survival time, which was still not reached by the end of the study for patients with scores <3 (Figure 3d). The mortality rates for all patients in the validation cohort at 0.5, 1 and 2 years after PPM implantation were 6.9%, 15.7% and 26.4%, respectively (Figure 4b). For patients with scores ≥3, the mortality rates rose to 26.3%, 31.6% and 57.9% at 0.5, 1 and 2 years after PPM implantation, respectively. By contrast, for patients with scores <3, the mortality rates were 2.4%, 12.0% and 19.3% at 0.5, 1 and 2 years after PPM implantation, respectively. An ROC analysis of MELODY score efficacy for predicting 0.5-, 1- and 2-year mortality rates after PPM implantation showed a significant predictive area under the curve values of 0.78, 0.71 and 0.72, respectively (Table 4).

## 4. Discussion

This study investigated pre-PPM implantation evaluation of life expectancy for patients aged 80 and older. We derived and validated a novel risk score for stratifying all-cause mortality risk prior to PPM implantation. The main findings were as follows: (1) The 0.5-, 1- and 2-year all-cause mortality rates after PPM implantation in patients aged 80 and older were 7%, 10–16% and 25%, respectively. Of the deceased patients, 74% died of non-cardiac causes during follow-up. No PPM implantation-related death occurred. (2) Prior to PPM implantation, the MELODY score composed of BMI <21 kg/m^2^, eGFR < 30 mL/min/1.73 m, LOS-B > 7 days and dyspnea as the major presenting symptom identified patients with high risk of all-cause mortality. (3) Patients with MELODY scores ≥3 were associated with a subsequent 8-fold increased risk of death compared to those with MELODY scores <3. More than 25%, 30% and 50% of patients with MELODY scores ≥3 died within 0.5, 1 and 2 years of follow-up after PPM implantation, respectively. Their median survival time after PPM implantation was less than two years. (4) For patients with MELODY scores <3, only 2–3%, 6–12% and 15–19% of them died after PPM implantation within 0.5, 1 and 2 years of follow-up, respectively. More than 80% of these patients survived longer than two years following the procedure. Their median survival time after PPM implantation was nearly seven years. The survival time is close to the mean time from initial PPM implantation to generator replacement in a previous study [13]. (5) The power of the MELODY score was validated in another cohort with notably different baseline characteristics.

Most previous studies of patients aged 80 and older receiving permanent pacemakers have focused on implant complications [14], prognostic factors and long-term outcomes [7,8,10]. There were rare studies about tools for evaluating life expectancy before PPM implantation. One study found that comprehensive geriatric assessment was useful in finding functional deficits that were associated with an increased risk of mortality after PPM implantation among patients aged ≥75 [15]. Balla et al. studied prognosis after pacemaker implantation in patients aged 80 and older and created a mortality risk prediction score consisting of various comorbidities and the use of a single-chamber device to identify high-risk old age patients at three months post-implantation [9]. In this study, we included patients’ clinical characteristics as potential items for analysis for the purpose of multifaceted and quick assessment at the bedside prior to PPM implantation. The developed scoring system stratified patients at high risk of mortality not only at 6 months but also at 1 and 2 years post-implantation. This scoring system offers an effective and easily available tool for evaluating short- and long-term mortality prior to PPM implantation in people aged 80 and older.

Similar to our results, prior studies have shown that more than 10% and 20% of patients aged 80 and older who received PPM implantation die of non-cardiac causes within 1 and 2 years, respectively, after PPM implantation [6,8,9]. With the aid of the scoring system, we further distinguished high-mortality risk patients among these extremely old patients. Since more than 25%, 30% and 50% of patients with scores ≥3 died within 0.5, 1 and 2 years after PPM implantation, respectively, early identification of these patients and further interventions like better nutritional support and rehabilitative programs should be considered to improve survival. For certain frail patients with atrioventricular block who have significant comorbidities that are likely to determine clinical outcomes, single-chamber ventricular instead of dual-chamber pacing could be considered [2]. Meanwhile, it is essential to aggressively optimize the PPM program setting during follow-up in order to enhance PPM generator longevity since more than 80% of patients with scores <3 survived longer than 2 years, and their median survival time after PPM implantation was up to 7 years, which is similar to the time from initial PPM implantation to generator replacement in former studies [13].

## 5. Study Limitations

First, functional assessments play an important role in the evaluation of life expectancy, but we did not include them due to the limited data available through chart review. Second, the scoring system was derived from a single hospital, and we could not exclude selection bias. To further prove the predictive power of the MELODY score, we validated it in another cohort with notably different baseline characteristics. Third, the retrospective design of this study limited our ability to make causal associations. Nevertheless, the prognosis factors in our study are rational since previous studies have identified them as significant risk predictors for mortality in certain populations [16,17,18,19,20,21,22,23]. Last, we excluded patients who received leadless pacemaker implantation. Previous studies demonstrated its safety and efficacy in comparison with transvenous single-chamber ventricular pacing [24] and in high-risk patients after transvenous lead extraction [25]. Further trials will be necessary to confirm our study results.

## 6. Conclusions

We demonstrated that the great majority of patients aged 80 and older tolerated PPM implantation and survived well after the procedure. Major complications were rare, and no PPM-implantation-related deaths occurred. Within 0.5, 1 and 2 years after PPM implantation, however, more than 5%, 10% and 20% of them died, respectively. More than 70% of deceased patients died of non-cardiac causes during follow-up. The developed and validated MELODY score, which is composed of BMI < 21 kg/m^2^, eGFR < 30 mL/min/1.73 m^2^, LOS-B > 7 days and dyspnea as the major presenting symptom, represents a simple and quick bedside tool to aid in all-cause mortality risk stratification prior to implanting PPM. In addition to PPM implantation, for patients with MELODY scores <3, physicians should aggressively optimize the PPM program setting to enhance PPM generator longevity because half of them survived nearly seven years after PPM implantation. For patients with MELODY scores ≥3, further interventions like nutritional support or rehabilitative programs should be considered to improve survival.

## Figures and Tables

**Table 1:** Baseline characteristics of patients aged 80 and older receiving permanent pacemaker implantation in the derivation and validation cohorts.

Characteristics	Derivation Cohortvb ^‡^	Validation Cohort	*p* Value
	(*n* = 100)	(*n* = 102)	
Age at implantation (years) *	84.5 (81.3–88.0)	85.0 (82.0–88.3)	0.73
Male gender, *n* (%)	52 (52.0)	38 (37.3)	0.04
Admitted from ED, *n* (%)	72 (72.0)	61 (59.8)	0.07
Left ventricle ejection fraction (%) *	68 (62; 73)	69 (62; 76)	0.47
Main symptoms at presentation			<0.001
Syncope, near syncope, *n* (%)	24 (24.0)	17 (16.7)	
Dizziness, *n* (%)	26 (26.0)	52 (51.0)	
Dyspnea, *n* (%)	50 (50.0)	33 (32.4)	
Comorbidities			
Hypertension, *n* (%)	88 (88.0)	77 (75.5)	0.02
Diabetes mellitus, *n* (%)	33 (33.0)	38 (37.3)	0.46
Coronary artery disease, *n* (%)	25 (25.0)	31 (30.4)	0.39
Valvular heart disease, *n* (%)	24 (24.0)	30 (29.4)	0.39
Cerebral vascular accident, *n* (%)	23 (23.0)	14 (13.7)	0.08
COPD, *n* (%)	19 (19.0)	17 (16.7)	0.67
Atrial arrhythmia, *n* (%)	39 (39.0)	54 (52.9)	0.04
eGFR (ml/min/1.73 m^2^) *	56.3 (32.6; 75.1)	51.0 (31.5; 72.4)	0.29
<30, *n* (%)	20 (20.0)	25 (24.5)	0.44
BMI (kg/m^2^) ^†^	24.0 ± 4.1	23.5 ± 4.6	0.39
<21, *n* (%)	17 (17.0)	31 (30.4)	0.03
AVCD, *n* (%)	70 (70.0)	43 (42.2)	<0.001
LOS-B (days) ^†^	6 (3;11)	3 (1;7)	0.001
Dual chamber pacemaker, *n* (%)	80 (80.0)	93 (91.2)	0.02
Follow-up duration (years) ^†^	4.0 ± 2.7	2.8 ± 1.3	<0.001

* Medians with interquartile range; ^†^ mean ± SD. AVCD, atrioventricular conduction dysfunction as the indication for pacemaker implantation; BMI, body mass index; COPD, chronic obstructive pulmonary disease; ED, emergency department; eGFR, estimated glomerular filtration rate; LOS-B, length of hospital stay before permanent pacemaker implantation. ^‡^ The details of derivation cohort were described in *Aging Clin Exp Res.* July 2019; 31(7): 1001–1009 [10], Springer Nature, adapted with permission.

**Table 2:** Univariate and multivariable analysis for predictors of all-cause mortality in patients aged 80 and older who received permanent pacemaker implantation in the derivation ‡ cohort.

Variables	Univariate Analysis		Multivariable Analysis		
	HR (95% CI)	*p* Value	Beta (SE)	HR (95% CI)	*p* Value
Age at implantation *	1.05 (0.99–1.11)	0.10			
Male gender	0.73 (0.43–1.24)	0.25			
Admitted from ED	1.22 (0.67–2.25)	0.52			
Ejection fraction (%) ^†^	0.99 (0.97–1.02)	0.66			
Dyspnea	1.73 (1.01–2.98)	0.04	0.64 (0.31)	1.90 (1.03–3.50)	0.03
Hypertension	1.03 (0.50–2.14)	0.93			
Diabetes mellitus	1.37 (0.78–2.42)	0.28			
Coronary artery disease	1.29 (0.69–2.43)	0.43			
Valvular heart disease	1.47 (0.81–2.67)	0.21			
Cerebral vascular accident	1.34 (0.74–2.44)	0.34			
COPD	1.07 (0.52–2.19)	0.87			
Atrial arrhythmia	0.79 (0.45–1.39)	0.42			
eGFR < 30 mL/min/1.73 m^2 †^	2.57 (1.40–4.72)	0.002	1.21 (0.32)	3.35 (1.77–6.35)	<0.001
BMI < 21 kg/m^2^	2.27 (1.14–4.49)	0.01	0.79 (0.37)	2.21 (1.06–4.61)	0.03
AVCD	2.07 (1.07–4.04)	0.03			
LOS-B > 7 days	2.32 (1.33–4.03)	0.003	0.63 (0.31)	1.87 (1.02–3.43)	0.04

* Per one-year increase in age. ^†^ Per unit increase. AVCD, atrioventricular conduction dysfunction; BMI, body mass index; CI, confidence interval; COPD, chronic obstructive pulmonary disease; ED, emergency department; eGFR, estimated glomerular filtration rate; HR, hazard ratio; LOS-B, length of hospital stay before pacemaker implantation; SE, standard error. ‡ The details of derivation cohort were described in *Aging Clin Exp Res.* July 2019; 31(7): 1001–1009 [10], Springer Nature, adapted with permission.

**Table 3:** Elements and their points contributing to the MELODY score for predicting all-cause mortality prior to permanent pacemaker implantation in patients aged 80 and older.

Characteristics	Point
B**M**I < 21 kg/m^2^	1
**e**GFR < 30 mL/min/1.73 m^2^	2
**LO**S-B > 7 days	1
**Dy**spnea as the main symptom at presentation	1

BMI, body mass index; eGFR, estimated glomerular filtration rate; LOS-B, length of hospital stay before pacemaker implantation.

**Table 4:** The areas under the receiver operating characteristic curves in predicting 0.5, 1 and 2-year all-cause mortality prior to permanent pacemaker implantation in patients aged 80 and older.

Cohort	0.5 Year		1 Year		2 Years	
	AUC	*p* Value	AUC	*p* Value	AUC	*p* Value
Derivation cohort	0.86	0.002	0.81	0.001	0.74	<0.001
Validation cohort	0.78	0.02	0.71	0.01	0.72	0.001

AUC, area under the receiver operating characteristic curve.

## Data Availability

The data that support the findings of this study are available from the corresponding author upon reasonable request.
